# Correspondence

**Published:** 1876-09

**Authors:** I. N. Danforth

**Affiliations:** Chicago


### Correspondence.

[The following communication was received too late for insertion in its proper place.—Ed.]

### REPLY TO DR. B. C. MILLER.

Messrs. Editors: It is rather difficult to frame a satisfactory reply to such a tangle of blunders as I find in the letter of Dr. B. C. Miller, published in your last issue. Nevertheless as the Doctor aims his blunderbuss mainly at me, I will with your permission, reply as briefly as possible.

Divested of its verbiage and translated into English, the Doctor's letter seems to mean, (1st.) That he was the originator of my investigations in relation to cholera in 1873, and that the work was carried on under the supervision of the Board of Health, whereof he was then Superintendent. (2d.) That the Board of Health "paid all expenses" connected with these investigations; and this proposition is so put as to lead your readers to believe that I was paid for the work. (3d.) That I have been guilty of discourtesy toward Dr. Ely McClellan, of the U. S. Army.

In support of the first proposition Dr. Miller cites the appointment of Dr. Simons as Assistant Sanitary Inspector, and the object of his appointment in the following language: "Dr. Simons was appointed Assistant Sanitary Inspector, with instructions to investigate fully the causes of the disease, and to make *post mortem* examinations of all cases, where one could be obtained, as I desired to have an examination made of the specimens obtained." (*Journal & Examiner*, for Aug.)

But on the 31st of March, 1874, in his *official* report, Dr. Miller writes as follows, concerning Dr. Simons' duties: "August 10th, (1873,) upon the retirement of Dr. Rauch, I was appointed Sanitary Superintendent, and visited the district" (*i. e.* the cholera district,) "for the first time in company with Dr. Simons, a physician residing in the district, and visited some ten or twelve cases. Dr. Simons was appointed *Special* Sanitary Inspector, with directions *to superin-*

*tend the enforcement of needed sanitary measures in that district, especially in the matter of daily disinfection, which was rigidly enforced on all premises.*" * (Italics are mine.)

I find the same declaration in regard to Dr. Simons, both in the "Report of the American Public Health Association," Vol. I., page 263, and also in the "Government Report on Cholera." † In his paper in the Government Report, Dr. Miller says, "Dr. Simons was appointed a *Special* Sanitary Inspector *to enforce the sanitary regulations of the district.*" (Italics are mine.)

It is scarcely necessary to call attention to the inconsistency of these two sets of "instructions," or to the fact that it was impossible for Dr. Simons to execute both; but it is proper to note that in his *official report*, *made more than two years ago*, Dr. Miller declares that Dr. Simons was charged with the "enforcement of sanitary measures," disinfection, etc., but he was *not* directed to "investigate the causes of the disease," or make *post mortems.* The conclusion is inevitable that in Dr. Miller's recent letter the instructions to Dr. Simons were revised to fit the present aspect of the case.

Dr. Miller states in several places that my examinations were made *at his request.* In this he is entirely mistaken. Before he made any "request" at all of me, my specimens were prepared and mounted, and my report partly written. *Then* he "requested" me to furnish my report, or an abstract thereof, that he might present it to the "American Public Health Association," then about to convene in New York.‡ With the approval of the then Secretary of the Chicago Society of Physicians and Surgeons, I did so; the paper was read before the Association, and published in its "Transactions" properly credited to me. On the evening of February 9th,

* Report of the Board of Health of the City of Chicago, for 1870–71–72–73, page 26.

† Cholera Epidemic of 1873, in the United States, page 215.

‡ This Association met about November 10th, 1873, (its officers were elected on the 13th,) whereas the Committee of the Society of Physicians and Surgeons was appointed August 18th and my investigations were commenced August 28th.

1874, I presented my completed report to the Society of Physicians and Surgeons. The meeting was quite fully attended, and I think Dr. Miller was present. The Society passed a vote of thanks for the report, and at the same time voted that the same be published in some medical journal. Some time after this Dr. Miller called upon me at my residence, and asked for the report that he might incorporate it in the Board of Health Report, then in course of preparation. At that time Dr. Miller *first* intimated to me that he was the real prime mover of all the investigations, and that he could rightfully claim my report. I remember that I felt and *expressed* some surprise, and I remember also that I felt considerable incredulity that I did *not* express. But as I cared little where the report was published, and as I regarded the vote of the Society of Physicians and Surgeons, above alluded to, as complimentary rather than mandatory, I yielded to the Doctor's solicitations, and allowed him to take that portion of the manuscript *which contained the record of my observations.* The first five or six manuscript pages he did *not* take. These pages contained the history of the origin of the investigations merely, and were of no scientific importance. Supposing, as a matter of course, that Dr. B. C. Miller, an honorable gentleman, and Sanitary Superintendent of the City of Chicago, could be trusted to say what was fair, true and right, and knowing as I did, that my preliminary remarks were needlessly prolix, especially for publication in a public report, I permitted the Doctor to omit the introductory pages, *as I wrote them*, *because* I believed that he would in fewer words place the *credit for the prosecution of the work* with the Society of Physicians and Surgeons, whatever he might say of himself as to originating the *idea* of the investigations. The proofs of that portion of my report which was published I saw; the proofs of Dr. Miller's remarks introductory thereto I did *not* see. Dr. Miller's official report, in its completed form, (of which mine formed a part,) I first saw after it was *bound and ready for circulation.*

In all this I am well aware that I have laid myself liable to criticism. I have only to say, however, that I did not deem it necessary to prescribe conditions that the Sanitary Superintendent of Chicago should do what common courtesy demanded, or institute a system of *espionage* to prevent what I did not fear or suspect.

*Secondly*—Dr. Miller asserts that the Health Department "paid all expenses connected with the examination," and this statement is repeated in a manner so loosely significant as to convey the impression that the Board of Health actually employed me to make investigations, and paid me for it. The "expenses connected with the examination" were merely nominal; but they were paid out of my own pocket, and the money has never been refunded by any one. It was, in fact, a matter so trivial that I have never thought it worth mentioning. As to the plates of which the Board of Health "retains possession," they consist of six small wood cuts; they were paid for, I suppose, by the City of Chicago. They are in the "possession" of the Bulletin Printing Company of this city, (or were at a very recent date,) whereof Dr. B. C. Miller is a thrifty partner. As the Health Report was printed by the Bulletin Printing Company, I suppose the wood cuts were left in their vaults for safe keeping. Believing that they might be of some use to me and of no use to the City of Chicago, I asked Dr. Miller for them in September last. He gave me an order on the Bulletin Printing Company for them, on condition that I would return them after using them; but the order was accompanied by a verbal admonition that if I purposed using them on my own account I ought to get my printing done at the Bulletin Printing Company. I still hold the order, and said company probably "retain possession" of the plates.

And this is the real story concerning the "pay," so far as I am concerned.

*Thirdly*—I regret exceedingly that Dr. McClellan's name has been dragged into this business. It is useless for Dr. Miller to try to divert attention from the real issue by making Dr. McClellan "party defendant;" besides, it is, to say the least, something less than magnanimous. In our article in the May (*not* June) number of this Journal, it never occurred to either Dr. Hyde or myself that we were complaining of Dr. McClellan, and certainly nothing could have been further from our intentions. For myself, I desire to say in the most positive terms, that Dr. McClellan's attitude toward me has so far as I know, been one of entire courtesy and liberality. That there may be no possibility of misunderstanding in the future I take this occasion to say that Dr. Hyde and I entertain the belief that Dr. B. C. Miller is the one person, and the *only* person, in fault as to the misappropriation of the work done by the Chicago Society of Physicians and Surgeons; except in so far as blame may attach to me personally, in that I trusted Dr. Miller too far. During the prosecution of his investigations in regard to the cholera epidemic of 1873, Dr. McClellan visited Chicago, and addressed the Society of Physicians and Surgeons, by special invitation. At the close of his remarks I was introduced to him, as were many of the members of the Society, and I remember that he spoke of my report in a complimentary manner. I *now* have a very indistinct recollection, also, that he intimated a desire to use my material in *his* report, then in course of preparation, although this fact had quite escaped my memory, until this recent discussion served to recall it. Exactly what passed between Dr. McClellan and myself, I do not now remember; but I believe that I went away with the understanding that I had been politely invited to furnish my report for the Government Report, but that nothing definite had been said on either side. I remember also, that I felt a little surprised because I did not subsequently hear from Dr. McClellan, on the subject. According to a letter from Dr. McClellan to Dr. Miller, the former wrote me "several times on the subject," but I have never received a letter, or a line, from Dr. McClellan in my life, on this or any other subject.

As a matter of fact, I think my investigations received more credit than they deserved, but however valuable or valueless they may be, they were *not* undertaken or completed at the "request" of Dr. B. C. Miller; he was *not* in "constant communication" or any other kind of "communication" with me while my studies were in progress; he never paid me for making the investigations, directly or indirectly, and, in fine, he never had anything to do with them from first to last.

The silly personalities in which Dr. Miller indulges, I pass by, because it is too small business for a man who has anything else to do, to notice them.

I. N. DANFORTH.

Chicago, August 5, 1876.

